# Cathodal Transcranial Direct Current Stimulation (tDCS) Applied to the Left Premotor Cortex Interferes with Explicit Reproduction of a Motor Sequence

**DOI:** 10.3390/brainsci11020207

**Published:** 2021-02-09

**Authors:** Bettina Pollok, Claire Schmitz-Justen, Vanessa Krause

**Affiliations:** 1 Institute of Clinical Neuroscience and Medical Psychology, Medical Faculty and University Hospital Düsseldorf, Heinrich-Heine University Duesseldorf, 40225 Duesseldorf, Germany; claire.schmitz-justen@hhu.de (C.S.-J.); vanessa.krause@hhu.de (V.K.); 2 Department of Neuropsychology, Mauritius Hospital and Neurorehabilitation Center Meerbusch, 40670 Meerbusch, Germany

**Keywords:** consolidation, motor sequence learning, non-invasive brain stimulation, SRTT

## Abstract

Transcranial direct current stimulation (tDCS) is a non-invasive brain stimulation technique that allows the modulation of cortical excitability. TDCS effects can outlast the stimulation period presumably due to changes of GABA concentration which play a critical role in use-dependent plasticity. Consequently, tDCS and learning-related synaptic plasticity are assumed to share common mechanisms. Motor sequence learning has been related to activation changes within a cortico-subcortical network and findings from a meta-analysis point towards a core network comprising the cerebellum as well as the primary motor (M1) and the dorsolateral premotor cortex (dPMC). The latter has been particularly related to explicit motor learning by means of brain imaging techniques. We here test whether tDCS applied to the left dPMC affects the acquisition and reproduction of an explicitly learned motor sequence. To this end, 18 healthy volunteers received anodal, cathodal and sham tDCS to the left dPMC and were then trained on a serial reaction time task (SRTT) with their right hand. Immediately after the training and after overnight sleep, reproduction of the learned sequence was tested by means of reaction times as well as explicit recall. Regression analyses suggest that following cathodal tDCS reaction times at the end of the SRTT training-block explained a significant proportion of the number of correctly reported sequence items after overnight sleep. The present data suggest the left premotor cortex as one possible target for the application of non-invasive brain stimulation techniques in explicit motor sequence learning with the right hand.

## 1. Introduction

The ability to learn and retain movement sequences is crucial for numerous everyday actions. Even seemingly easy movements, like grasping an object, require the well-coordinated execution of distinct elements that are combined to a coherent motor sequence. The most common paradigm for the investigation of motor sequence learning is the serial reaction time task (SRTT) originally introduced by Nissen and Bullemer [1]. In this task, the participants learn a sequence of keypresses in response to visual cues. Learning is indicated by reduced errors as well as shortening of reaction times during training on the task and can occur explicitly by informing the participants about the embedded sequence or implicitly through repeated practice (reviewed in [2,3,4,5,6,7]). Besides acquisition, the term motor learning comprises the consolidation of the previously learned motor skill indicated by either reduced susceptibility to an interfering pattern (i.e., skill stabilization) or further performance enhancement after and between training sessions (i.e., offline improvement; for example, [8]; reviewed in [4,5,7,9,10,11,12]). Consolidation allows the transfer of an initially fragile movement pattern into a more robust state [13,14]; reviewed in [4,5,7,9,10,11,15,16] and involves alterations on the level of synapses and neural networks as well as changes of the neuronal protein synthesis (reviewed in [11,17]). Motor skill consolidation particularly in explicit learning tasks benefits from sleep (reviewed in, e.g., [18]). In contrast to this, in implicit tasks consolidation appears to depend on the passage of time rather than sleep [19]; reviewed in [4,5,7,11,18].

Functional neuroimaging studies have been widely used to determine the neural network underlying motor learning (e.g., [20,21,22,23,24,25,26,27,28,29]; reviewed in [3,4,30,31]; for meta-analysis refer to [32,33]). Those data suggest the involvement of prefrontal, primary and premotor as well as parietal areas, the cerebellum and the basal ganglia. Motor sequence learning by means of the SRTT with the right hand has been found to consistently engage bilateral dorsolateral premotor cortices (dPMC) and supplementary motor areas (SMA), the ventral PMC of the right hemisphere as well as the primary motor cortex (M1), the superior parietal cortex (SPC) and the thalamus of the left hemisphere and the right cerebellum (e.g., [32]). Although evidence for a bi-hemispheric network exists (e.g., [22,31]), a core network subserving motor learning has been identified including particularly the left dPMC [32], which seems to be engaged in early phases of motor learning [25,34,35]; for meta-analysis refer to [33] and evidence for learning-induced microstructural changes within the dPMC exists [22]. The initial strong activation might reflect the use of explicit learning strategies [36]. This interpretation is supported by the analysis of resting state networks suggesting that functional connectivity distinctively changes in implicit and explicit task variants across a six hours consolidation period [37,38].

Although a substantial overlap of areas engaged in explicit and implicit learning of the SRTT has been suggested [39] evidence for at least partly distinct networks exists [37,40,41]; reviewed in [6,10,11]. Explicit variants of the SRTT are stronger associated with activation of a fronto-parietal network [37]; reviewed in [5], which was found to correlate with the correct recall of the previously learned sequence [40]. In contrast to this, implicit learning appears to be associated with increased activation of the contralateral primary sensorimotor cortex [40]. Findings from a meta-analysis support the involvement of bilateral premotor areas as well as the left superior posterior parietal cortex in explicit as compared to implicit task variants of the SRTT [32] and evidence exists that PMC activation increased as the participants became aware of the sequential nature of the executed movement [26] or when movements are directed by explicit information [36].

Transcranial direct current stimulation (tDCS) is a non-invasive brain-stimulation technique in which low-intensity constant currents are applied to the brain via scalp electrodes. The basic idea behind this method is that weak currents modulate neuronal resting membrane potentials in a polarity specific manner: While anodal tDCS exerts a constant depolarization thereby facilitating spontaneous neuronal activity, cathodal tDCS yields hyperpolarization (reviewed in [42,43,44]). With longer stimulation intervals tDCS effects can outlast the stimulation period presumably due to N-methyl-D-aspartate (NMDA) receptor driven long-term potentiation (LTP) and long-term depression- (LTD) like plasticity of the cortex (reviewed in [42,43]). Anodal tDCS applied to M1 was shown to be associated with reduced GABA levels [45], while cathodal tDCS went along with alterations in GABA as well as Glutamate concentration [45]. Changes of GABA concentration play a critical role in use-dependent plasticity within M1 [46] and reduced levels in M1 following application of anodal tDCS have been shown to be correlated with improved motor performance following practice [47]. Accordingly, tDCS-induced GABA concentration changes can predict individual differences in motor learning [48]. Thus, anodal tDCS and motor-learning may share common mechanisms and improved motor learning associated with anodal tDCS applied to M1 [49,50,51,52,53]; reviewed in [9,54,55] may occur due to additive effects of learning- and tDCS-induced neuroplasticity. Importantly, such effects were found when stimulating the M1 and the effect of tDCS (excitatory vs. inhibitory) strongly depends on the direction of current flow in the brain which may vary between individuals due to anatomical differences and likely between brain areas (reviewed in [56]). Furthermore, one of the limitations of this type of non-invasive brain stimulation is the relatively low spatial focality (reviewed for example, in [9,57]).

Despite such methodological weakness, previous studies applying non-invasive brain stimulation techniques to the premotor cortex (PMC) suggest effects on retention tests following training on continuous tracking tasks [58,59] as well as motor sequences [60,61,62]. Those findings suggest the dPMC’s involvement in motor consolidation in implicit learning paradigms. The present study aims at investigating its functional role in an explicit task variant. Thus, tDCS was applied immediately prior to SRTT training with the aim to “prime” the motor system for subsequent motor sequence learning according to [58,60,61]. Since memory retrieval allows the assessment of encoding and consolidation of new information and skills (reviewed in [63]), reaction times as well as reproduction of the previously trained sequence were determined immediately after training on the task and after overnight sleep. Assuming that the dPMC is involved in explicit motor learning we hypothesize that cathodal tDCS should interfere with explicit sequence learning while a facilitating effect following anodal tDCS is expected. These effects should be particularly evident after overnight sleep due to the beneficial effect of sleep on motor skill consolidation in explicit sequence learning (reviewed in [18]).

## 2. Materials and Methods

Eighteen healthy volunteers (9 female) participated in the study. Mean age was 24.83 ± 0.89 years (mean ± standard error of the mean (S.E.M.)) and varied between 19 and 32 years. The sample size was chosen since we expected an effect size comparable to a previous study in which tDCS was applied to the left dPMC in an implicit task variant [61]. The participants had normal or corrected to normal vision and none of them reported health impairment. Handedness was tested by means of the Edinburgh Handedness Inventory (EHI) [64] (for results please refer to the section number 3). Participants with individual or family history of epileptic seizures or other neurological, psychiatric or internal diseases—in particular, cardiac pacemaker—and intake of central nervous system-active medication were excluded from study participation. The health condition was ascertained by the participants’ self-reports [61]. In addition, in case pregnancy was not excluded, volunteers were not considered for study participation. Written informed consent was provided individually prior to the first experimental session. The study is in line with the latest version of the Declaration of Helsinki and was approved by the local ethics committee of the medical faculty of the Heinrich-Heine University (study number 3347).

### 2.1. Transcranial Direct Current Stimulation (tDCS)

The left dPMC was localized as the stimulation target by means of neuronavigation (LOCALITE, Sankt Augustin, Germany) with Talairach coordinates −29, 5, 47 (*x*, *y*, *z*) corresponding to Brodmann area 6. Anatomical landmarks (i.e., bilateral pre-auricular points, nasion, inion and the surface of the skull) were localized in each individual and were transformed onto a standard brain. We additionally localized the cortical representation of the right first dorsal interosseus (FDI) muscle using single pulse transcranial magnetic stimulation (TMS) to ensure that the active electrode does not overlap with M1. A standard figure of eight coil (MC-B70) connected to a MagPro stimulator (Mag Venture, Hueckelhoven, Germany) was applied to trigger motor evoked potentials (MEPs). The coil was placed tangentially to the scalp with the handle pointing in posterior-lateral position at an angle of 45° and was moved in 0.5 cm steps anterior, posterior, medial and lateral in order to determine the area with the largest MEP amplitude. The left hemisphere was chosen as target for tDCS since evidence for a stronger involvement of the left cortical hemisphere for the execution of sequential movements comprising more than one finger has been shown [65].

**Figure 1:**
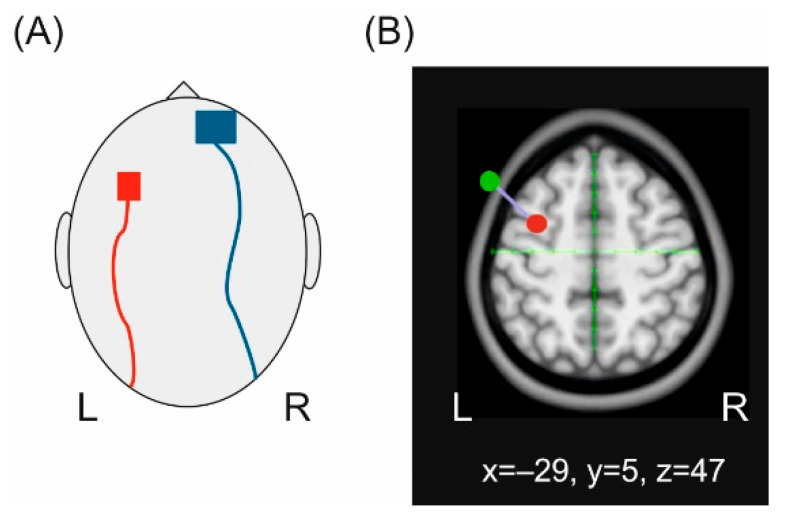
Transcranial direct current stimulation (tDCS). (**A**) Schematic illustration of the electrode positions. (**B**) Illustration of the tDCS-target area projected onto a standard brain. The red dot indicates the area to be stimulated corresponding to left BA 6, the green dot the localization of the active electrode attached to the head.

TDCS was applied by means of two rubber electrodes nested in saline soaked sponges. Prior to stimulation, the skin resistance was decreased by degreasing the skin using an abrasive paste and ethanol. The electrodes were applied using a standard montage [66] with the active electrode (3 × 3 cm^2^) placed over the left dPMC while the return electrode (5 × 5 cm^2^) was placed over the right orbit. A larger return electrode was chosen to minimize prefrontal co-stimulation [67] and to increase the focality of dPMC stimulation (reviewed in [68]) [69]. Both electrodes were fixed with self-adhesive elastic bandages (Coban, 3M Deutschland GmbH, Neuss, Germany). The electrode cables were applied in an anterior-posterior direction. The electrode montage as well as the localization of the target area are schematically illustrated in Figure 1.

A battery-driven direct current stimulator (DC-Stimulator Plus, Eldith, Neuroconn, Ilmenau, Germany) was used for tDCS with an intensity of 0.25 mA yielding 0.03 mA/cm^2^ average current density below the active electrode and 0.01 mA/cm^2^ below the return electrode. This intensity was chosen since an average current density of approximately 0.028 mA/cm^2^ below the active electrode has been shown to significantly modulate implicit motor sequence learning when applied to M1 [50] as well as to PMC [61]; for a review refer to [9]. During anodal and cathodal stimulation tDCS was applied for ten minutes according to [61] and in line with previous studies (reviewed in [9]) with additional fade-in and fade-out periods of ten seconds, respectively [69]. Sham tDCS served as control condition [68,69] and was realized by a 30 s stimulation of either anodal or cathodal polarity with the same fade-in and fade-out periods and stimulation intensity as during real tDCS. This set-up induces the typical stimulation-associated sensations such as a slight tingling allowing blinding of the participants with respect to the stimulation type. For sham stimulation the polarity was counterbalanced across participants and experimental sessions. Impedance was kept below 10 kOhm and the DC-stimulator switched off automatically in each condition. After each experimental session, the participants estimated the stimulation condition by questionnaire to control for successful blinding. TDCS was applied with respect to safety guidelines [70,71,72] and possible stimulation related adverse effects were determined after each session by a modified questionnaire according to [71]. Finally, the electrical field strength was simulated using SimNIBS 3.2.2 [73]. Since individual magnetic resonance imaging (MRI) scans of the participants were not available, the included “Ernie” example data-set was applied which consists of high-resolution T1- and T2-weighted and diffusion MR images acquired with a 3.0 T Philips Achieva MR scanner. The data are from a young healthy male who gave his consent to the publication of his MRI scan which was fully anonymized including depersonalization of the facial region [73].

### 2.2. Explicit Motor Sequence Learning

**Figure 2:**
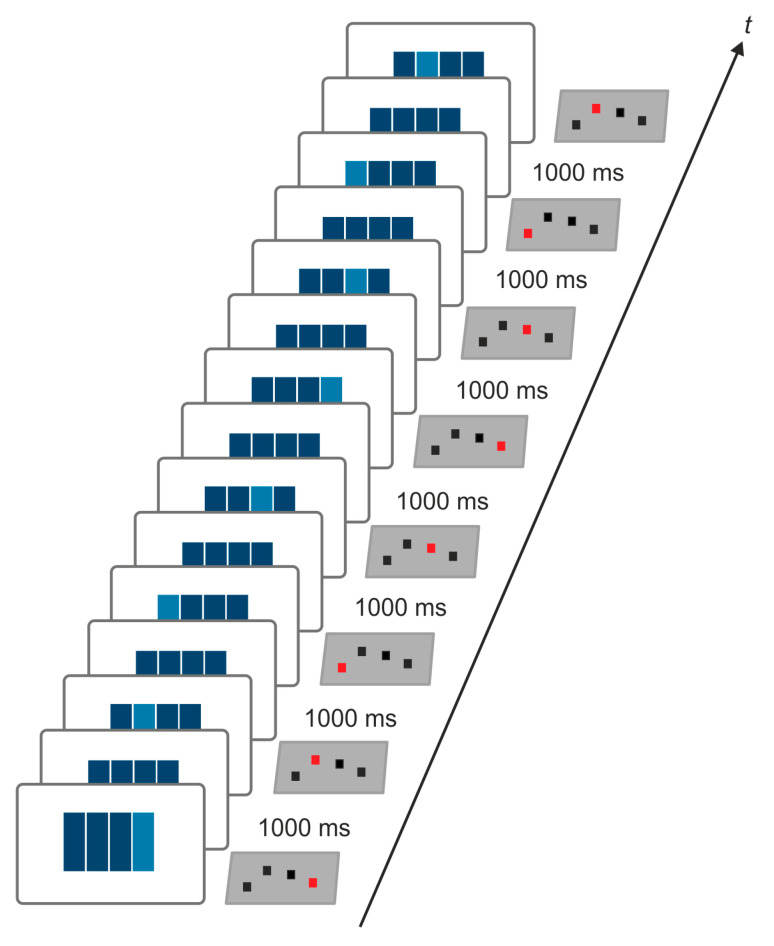
Serial reaction time task (SRTT). The SRTT is realized by four horizontally aligned bars presented on a screen in front of the participants. The figure exemplifies one of the three sequences applied in the present study. Each bar corresponded to one button of a custom-made response box, which was connected to a standard windows PC. The participants were instructed to respond as fast and as accurately as possible by button press with the right hand as soon as one bar changed its color. Each correct response was followed by a fixed interval of 1000 ms.

The SRTT [1] was applied to induce explicit learning of an eight-digit motor sequence. To this end, four dark blue horizontally aligned bars were presented on a screen in front of the participants (height: 0.9 m, width of the entire stimulus: 1.8 m, distance: 2.5 m) [61]. Each bar corresponded to one button of a custom-made response box aligned to the right hand. Four buttons corresponding to the first four fingers of the right hand were used for the experiment [61]. The button box was connected to a standard Windows-PC allowing collecting of button press onsets as a measure of reaction times. E-Prime (Psychology Software Tools Inc., Sharpsburg, PA, USA) was used for SRTT timing and recording of reaction times. The participants were instructed to press the correct button as fast and as correct as one of the four bars changed its color to light blue. After a fixed inter-stimulus interval of 1000 ms, the next bar changed the color upon correct response. In case of incorrect responses, the bar remained light blue until the correct button had been pressed [61,74]. Stimuli were presented either as an eight-digit fixed repeating sequence (sequential) or as eight randomly varying stimuli (random) with the latter serving as control condition for unspecific reaction time improvement due to practice with the apparatus [2]. The frequency of stimuli was kept constant in both conditions. The onset of each visual stimulus was aligned with the 60 Hz refresh rate of the monitor by means of 25% refresh alignment. In order to induce explicit motor sequence learning the participants were informed that the same sequence will be presented in a training block of 20 repetitions and that the order of button presses (i.e., the sequential pattern) will be queried immediately after the end of training and on the next day after overnight sleep. In order to minimize transfer effects between experimental sessions, three versions of the SRTT were used (sequence 1: 4-2-1-3-4-3-1-2; sequence 2: 3-4-2-1-2-4-3-1; sequence 3: 3-2-1-4-3-2-4-1) [61,74]. SRTT versions were counterbalanced across participants and stimulation conditions. In a previous pilot study (unpublished data) the versions were shown to yield comparable reaction time acceleration and were applied to induce implicit motor sequence learning in previous studies [61,74]. The repetition rate was also chosen due to findings from the mentioned pilot study suggesting reaction time improvement over the first 20 repetitions. After that reaction times became more variable and tended to increase. The SRTT is exemplified in Figure 2.

To test explicit sequence knowledge, the participants were asked to reproduce the trained sequence by means of button presses immediately after the training and after overnight sleep. The number of correctly reproduced items was counted for further analyses.

### 2.3. Design

A within-subjects, double-blind, sham-controlled design was employed for the present study. The participants were blind regarding the respective stimulation condition and the exact purpose of the study. None of them received tDCS before. To ensure blinding of the investigator regarding the tDCS conditions, a second investigator was responsible for handling the DC-stimulator [61,74]. The participants received anodal, cathodal and sham tDCS in three subsequent sessions. To avoid carry-over effects, sessions were separated by at least one week. The sessions were performed between nine a.m. and six p.m. and time of participation was held constant for each subject.

The participants were informed that the training block consists of a sequential pattern and they were instructed to identify the pattern during the training to induce explicit motor sequence learning. No additional practice sessions were given prior to baseline trials.

**Figure 3:**
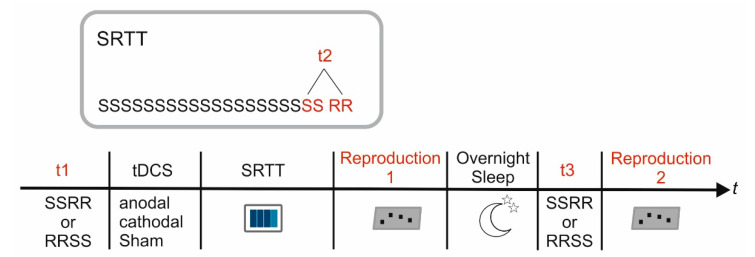
Study Design. Reaction times were analyzed at baseline prior to SRTT training (t1), at the end of the training block (t2) and after overnight sleep (t3). The insert indicates details of the SRTT: After 20 repetitions of the sequential pattern (S) 16 randomly varying stimuli (RR) were presented to estimate unspecific reaction time improvement. Explicit sequence knowledge was determined immediately after the end of SRTT training (reproduction 1) as well as after overnight sleep (reproduction 2).

Each session started with a baseline measurement (t1) consisting of two repetitions of the respective sequence and the same number of randomly varying stimuli (i.e., 16 button presses for each condition). The order of conditions (sequential vs. random) was counterbalanced across participants and stimulation conditions [61,74]. After that tDCS was applied during rest followed by training on the SRTT by means of 20 repetitions of the same sequence. Reaction times averaged across the last two sequences of the training block served as outcome measure for the estimation of motor sequence learning (t2_sequential_). The training block ended with two repetitions of eight randomly presented stimuli serving as control condition for unspecific reaction time improvement (t2_random_). Thereafter the participants were asked to reproduce the sequential pattern by means of button presses (reproduction 1). On the next day the experiment was continued by testing reaction times with respect to two repetitions of the previously learned sequence (t3_sequential_) and the same number of randomly varying stimuli (t3_random_). Finally, reproduction of the learned sequence was again tested by means of button presses (reproduction 2). Please note, that reproduction performance was tested once at each time point. The study design is illustrated in Figure 3.

### 2.4. Statistics

Reaction times in each condition were determined individually at time points t1–t3. In addition, reaction times during SRTT training were averaged across four subsequent repetitions of the sequence resulting in five bins to estimate motor sequence learning during training on the task. The number of correctly reproduced items was ascertained immediately after SRTT training as well as on the next day after collecting reaction times. The data were analyzed by means of IBM SPSS Statistics 25. In a first step the data were controlled for Gaussian distribution by means of Kolmogorov Smirnov goodness of fit test. For regression analyses Gaussian distribution of the residuals was ensured accordingly. The effect of tDCS on reaction times over the time course of SRTT training was analyzed by analysis of variance (ANOVA) with factors *time* (t1_sequential_ vs. bin 1 vs. bin 2 vs. bin 3 vs. bin 4 vs. bin 5) and *stimulation* (anodal vs. cathodal vs. sham). To ensure that the expected reaction time acceleration was specific to sequential trials reaction times at t1 and t2 were analyzed by ANOVA with factors *condition* (random vs. sequential), *time* (t1 vs. t2) and *stimulation* (anodal vs. cathodal vs. sham). Since a facilitating effect of anodal tDCS on task performance in a visuomotor paradigm has been observed at the beginning of the training [51], we additionally compared reaction times at t1 and the first bin with factors *time* (t1 vs. bin 1) and *stimulation* (anodal vs. cathodal vs. sham). To investigate the effect of tDCS on subsequent reaction times as a measure of motor sequence consolidation, ANOVA was calculated with factors *time* (t1 vs. t2 vs. t3), *condition* (random vs. sequential) and *stimulation* (anodal vs. cathodal vs. sham). The number of correctly reproduced sequence items was analyzed by means of ANOVA with factors *time* (reproduction 1 vs. reproduction 2) and *stimulation* (anodal vs. cathodal vs. sham). Finally, the relation between reaction times in sequential trials at t1 and t2 and reproduction performance as a measure of explicit sequence knowledge was estimated by regression analyses. *P*-values were corrected for multiple testing by means of the sequential Bonferroni correction [75]. Greenhouse-Geisser correction was applied whenever sphericity assumption was violated.

## 3. Results and Discussion

Mean lateralization ratio as revealed by EHI was 97.44 ± 1.85 suggesting that all participants were right-handed. The distance between M1 and the stimulation area was on average 3.7 ± 0.2 cm (anodal), 3.3 ± 0.2 cm (cathodal) and 3.7 ± 0.2 cm (sham). No significant difference between tDCS-conditions was found (*F*(2, 34) = 1.943, *p* = 0.159, *η*^2^ = 0.103). Mean impedance was 6.75 ± 0.39 kΩ and did not differ significantly between stimulation conditions (anodal: 6.63 ± 0.7 kΩ; cathodal: 6.79 ± 0.67 kΩ; sham: 6.82 ± 0.65 kΩ; (*F*(2, 34) = 0.057, *p* = 0.945, *η*^2^ = 0.003)).

**Figure 4:**
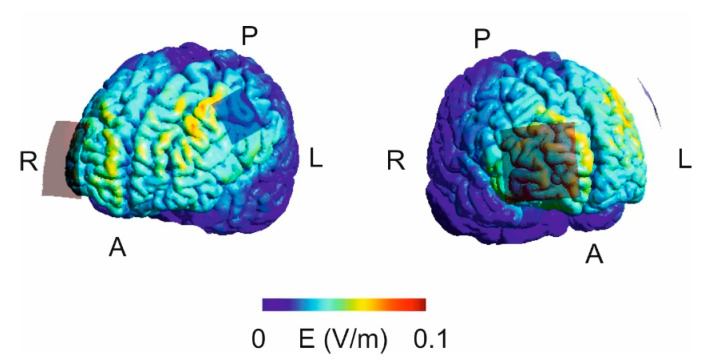
Visualization of the electrical field strength by means of SimNIBS and the included “Ernie” magnetic resonance imaging (MRI) data set. The analysis suggests a widespread stimulation effect with the strongest electrical field anterior to the active electrode (A = anterior; P = posterior; L = left; R = right).

Simulation of the electrical field by means of SimNIBS 3.2.2 and the included “Ernie” MRI-data set suggests a widespread stimulation effect on the frontal cortex. The strongest fields were evident anterior to the active electrode. Weaker fields were found below and mesial to the return electrode (Figure 4).

The analysis of the stimulation questionnaire indicated that tDCS was correctly identified in 33% (anodal tDCS and sham) and in 28% (cathodal tDCS) of all sessions suggesting that blinding of the participants regarding the tDCS conditions was successful. In 18 of 54 sessions the participants reported mild adverse effects of tDCS (Table 1). In none of the participants severe adverse effects were observed.

### 3.1. Reaction Times during SRTT Training

The analysis of reaction times during the time course of SRTT training suggests a significant main effect of factor *time* (*F*(2.5, 27.7) = 50.75, *p* < 0.000, *η*^2^ = 0.822), while neither factor *stimulation* (*F*(2, 22) = 0.153, *p* = 0.859, *η*^2^ = 0.014) nor the *time × stimulation* interaction (*F*(10, 110) = 1.60, *p* = 0.116, *η*^2^ = 0.127) were significant. The comparison of reaction times between random and sequential trials at t1 and t2 indicates significant main effects of factors *time* (*F*(1, 11) = 28.50, *p* < 0.000, *η*^2^ = 0.721) and *condition* (*F*(1, 11) = 67.78, *p* < 0.000, *η*^2^ = 0.860) as well as significant *time × stimulation* (*F*(2, 22) = 4.90, *p* = 0.017, *η*^2^ = 0.308) and *condition × time* (*F*(1, 11) = 69.37, *p* < 0.000, *η*^2^ = 0.863) interactions. All other comparisons did not reach significance (*p* > 0.526, *η*^2^ < 0.057). The *condition × time* interaction indicates significantly faster reaction times at t2 as compared to t1 in sequential trials (*t*(17) = 10.29, *p* < 0.000), while in random trials no significant differences between time points were evident (*t*(17) = −2.36, *p* = 0.09). Consequently, at t1 reaction times between random and sequential trials did not differ significantly from each other (*t*(17) = −1.59, *p* = 0.516), while at t2 reaction times were significantly faster in sequential as compared to random trials (*t*(17) = −13.21, *p* < 0.000).

**Figure 5:**
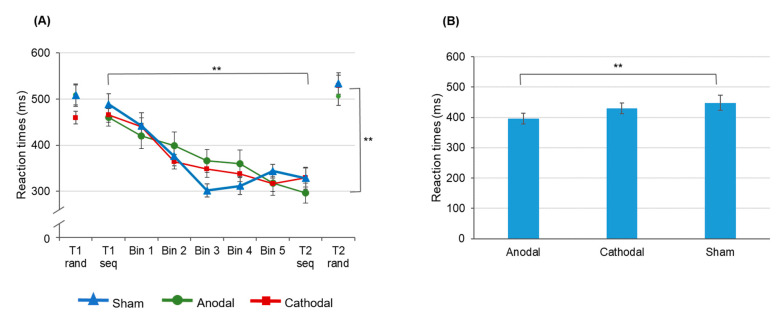
Changes of reaction times over the time course of SRTT training. (**A**) The results indicate significant acceleration of reaction times in sequential but not in random trials. (**B**) At t2 reaction times (averaged across random and sequential trials) were significantly faster following anodal tDCS as compared to sham stimulation. Error bars indicate the standard error of the mean (S.E.M.) (** *p* ≤ 0.01).

The *time × stimulation* interaction is due to faster reaction times at t2 following anodal tDCS (396.14 ± 18.41 ms) as compared to sham stimulation (448.06 ± 24. 81 ms; *t*(16) = −2.292, *p* = 0.010). Following cathodal tDCS reaction times at t2 were on average 429.32 ± 18.12 ms and did neither significantly differ from anodal tDCS (*t*(15) = −1.239, *p* = 0.234) nor from the sham condition (*t*(16) = 0.432, *p* = 0.671). Reaction time acceleration following anodal tDCS was found independent of the task (random vs. sequential) suggesting an unspecific stimulation effect. A comparable effect has been shown for the implicit task variant [61] and may occur due to an excitatory effect of anodal tDCS on corticospinal excitability as has been shown for TMS [76,77] yielding a beneficial effect on task performance. The data are summarized in Figure 5.

In a further step, we analyzed possible tDCS effects on reaction times at the beginning of SRTT training [51]. To this end, reaction times in sequential trials at t1 and the first bin of the training block were compared by means of ANOVA with factors *stimulation* (anodal vs. cathodal vs. sham) and *time* (t1 vs. bin 1). Again, this analysis did not provide evidence for significant tDCS effects as indicated by a non-significant main effect of factor *stimulation* (*F*(2, 24) = 0.724, *p* = 0.495, *η*^2^ = 0.057) and a non-significant *time × stimulation* interaction (*F*(2, 24) = 0.050, *p* = 0.951, *η*^2^ = 0.004). A trend towards significance of factor *time* was found (*F*(1, 12) = 3.656, *p* = 0.080, *η*^2^ = 0.234) suggesting a training effect occurring already within the first four repetitions of the sequence. The results from this analysis argue against short-lasting tDCS effects on reaction times during training on the SRTT.

All in all, the present data indicate that—despite the relatively low repetition rate during SRTT training—motor sequence learning as indicated by reaction time improvement in sequential but not in random trials was sufficiently well induced. Although an unspecific facilitating effect of anodal tDCS on reaction times was found, the data do not provide evidence for a significant dPMC tDCS effect on the acquisition of an explicitly learned motor sequence. This finding is in line with those from an implicit task variant [61].

### 3.2. Reaction Times after SRTT Training

**Figure 6:**
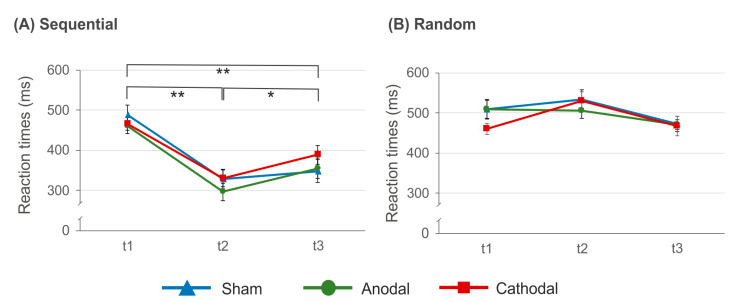
Reaction times at t1, t2 and t3 in the sequential (**A**) and random trials (**B**). In sequential but not in random trials a significant reaction time acceleration at t2 and t3 as compared to t1was found. This effect occurred independent of tDCS condition. Error bars indicate the standard error of the mean (S.E.M.) (* *p* ≤ 0.05; ** *p* ≤ 0.01).

The analysis of reaction times at t1, t2 and t3 suggests significant main effects of factors *condition* (*F*(1, 11) = 79.625, *p* < 0.000, *η*^2^ = 0.879) and *time* (*F*(2, 22) = 17.738, *p* < 0.000, *η*^2^ = 0.617) as well as a significant *condition × time* interaction (*F*(2, 22) = 52.004, *p* < 0.000, *η*^2^ = 0.825). The *time × stimulation* interaction just missed significance (*F*(2, 24) = 3.370, *p* = 0.051, *η*^2^ = 0.219). Since the above mentioned ANOVA including t1 and t2 only suggests this interaction to be significant, we additionally calculated the statistical power (i.e., 1 − β) of the comparison between all three time points with *α* = 0.05, *f* = 0.457, *r* = 0.1 and *N* = 18 by means of G*Power [78]. Calculation of the *f*-value is based on the data variance. The analysis indicated 1 − β = 0.959, suggesting the statistical power to be sufficiently high. All other comparisons were not significant (*p* > 0.074, *η*^2^ < 0.173). The *condition × time interaction* is due to significantly faster reaction times in sequential trials at t2 (321.91 ± 16.41 ms; *t*(17) = 10.293, *p* < 0.000) as well as at t3 (371.94 ± 23.88 ms; *t*(17) = 5.11, *p* < 0.000) as compared to t1 (472.56 ± 13.93 ms). Although at t3 reaction times were slower as compared to t2 (*t*(17) = −2.816, *p* = 0.012) the data indicate persisting learning effects. In contrast to this, in random trials neither reaction times at t2 (525.23 ± 17.04 ms; (*t*(17) = −2.361, *p* = 0.06) nor at t3 (472.65 ± 16.43 ms; *t*(17) = 1.21, *p* = 0.242) were significantly different from that at t1 (490.44 ± 15.10). The results are summarized in Figure 6.

### 3.3. Sequence Reproduction

On average, 7.1 sequence items were correctly reproduced at reproduction 1, while the number decreased to 6.8 after overnight sleep (reproduction 2). The ANOVA revealed a main effect of factor *time* (*F*(1, 17) = 8.511, *p* = 0.010, *η*^2^ = 0.334), while neither factor *stimulation* nor the *time × stimulation* interaction turned out to be significant (*p* < 0.465, *η*^2^ > 0.041). The high reproduction rate indicates that explicit learning was successfully induced and—despite the significant decrement after sleep—sequence knowledge was retained until the next day.

**Figure 7:**
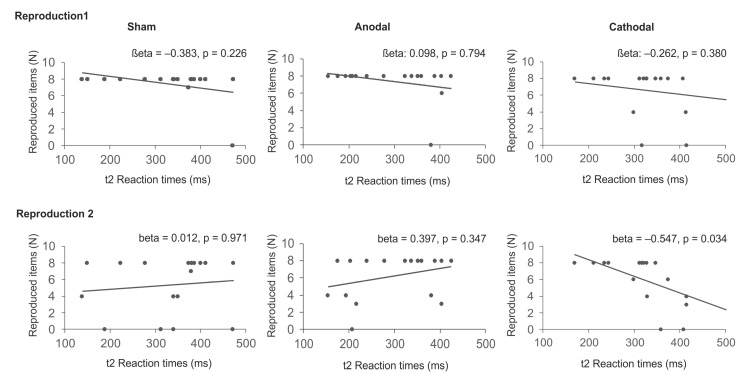
Regression analyses. Correlation between reaction times in sequential trials at t2 and the number of correctly reproduced items immediately after SRTT training (Reproduction 1) and after overnight sleep (Reproduction 2). The analyses suggest that following cathodal tDCS reaction times in sequential trials significantly predict explicit reproduction after sleep.

To estimate the relation between reaction times in sequential trials at t1 and t2 and the reproduction of sequence items, linear regression analyses were calculated for each stimulation condition with the number of reproduced items (reproduction 1 and reproduction 2) as dependent variables and reaction times in sequential trials at t1 and t2 as independent variables. Kolmogorov-Smirnov goodness of fit test ensured Gaussian distribution of the residuals (*p* = 0.330). The analyses suggest that following cathodal tDCS reaction times explained a significant proportion of the number of correctly reported sequence items at reproduction 2 (*R*^2^ = 0.410, *F*(2,12) = 4.166, *p* = 0.042) with reaction times in sequential trials at t2 significantly predicting reproduction performance (*beta* = −0.547, *p* = 0.034).) The remaining analyses did not reveal evidence for significant results (*p* > 0.152). The data are summarized in Table 2 and Figure 7.

To ensure that this effect is indeed due to reaction times in sequential but not in random trials, two control analyses were conducted for the cathodal condition with reproduction 2 as dependent variable. In the first calculation, reaction times in random trials at t1 and t2 were selected as independent variables. This analysis did not provide evidence for a significant effect (*R*^2^ = 0.267, *F*(2, 14) = 2.543, *p* = 0.114). In a second analysis reaction times in random and sequential trials at t2 were selected as independent variables suggesting a significant regression (*R*^2^ = 0.493, (*F*(2, 14) = 6.798, *p* = 0.009). While reaction times in sequential trials were again found to significantly predict reproduction performance after sleep (*beta* = −0.508, *p* = 0.023), reaction times in random trials did not (*beta* = −0.358, *p* = 0.094).

As the main finding, the present data imply that following cathodal tDCS reaction times at the end of the training block can predict the explicit reproduction rate of the learned sequence on the next day. The control analyses support the hypothesis that the effect of cathodal tDCS is specific to the learned sequence and cannot be attributed to a general slowing of reaction times following cathodal tDCS. Following anodal tDCS reaction times at t2 were significantly faster as compared to sham stimulation. But, this effect was not specific to sequential trials. The lack of showing a significant correlation between reaction times at t2 and reproduction performance might be due to a ceiling effect as the reproduction rate was high: 17 of 18 participants were able to correctly reproduce the entire sequence following anodal tDCS and sham stimulation. Vidoni and co-workers [36] suggest that the initial activation increase of the dPMC during motor learning might reflect the use of explicit learning strategies. Thus, reducing the excitability should yield a decline in explicit motor learning. The present findings support this assumption and are in line with the notion of a core motor-learning network comprising premotor regions [32] and support findings from previous tDCS [61,62,79] and TMS-studies [58,59] suggesting the relevance of the left dPMC for motor consolidation of implicitly learned tasks. Accordingly, activation changes within a posterior parietal-premotor network have been shown to correlate with behavioral improvement during training [20] and retention five days after the training [24]. In addition, evidence exists that sequences are represented in premotor and inferior parietal areas [80,81] and spatial and temporal features of a sequence appear to be separately stored in dPMC enabling flexible adjustments of a motor pattern as well as encoding of complex motor behaviors [82].

Nevertheless, the data do not agree with those from a previous study indicating no significant effect of dPMC-tDCS applied immediately after the training in a group of healthy older volunteers [83]. Although age differences between participants may have contributed to this inconsistency, it additionally points towards the significance of the brain state during stimulation (reviewed in [56]).

Notwithstanding that the present study supports the dPMC’s involvement in motor sequence learning, this notion has been challenged by a recent meta-analysis suggesting that particularly the basal ganglia directly contribute to motor sequence learning while premotor regions as well as the cerebellum do not [84]. Thus, it has been argued that increased activity in cerebellar and premotor regions during motor learning might reflect behavioral changes associated with motor learning rather than learning per se [28]. A recent study identified beta oscillations within a frontal-parietal network, including the PMC, to predict micro-offline gains in short resting periods between training sessions possibly reflecting reactivation of practice-related activity or memory replay [8] and neurons within the dPMC have been suggested to integrate sensory information with motor instructions facilitating higher order temporal organization of sequences (reviewed in [85]). Thus, behavioral improvement associated with training on a task may particularly occur due to non-motoric reasons like improved recall of the sequence order [6]. Those findings point towards the hypothesis that sequence learning occurs due to improved cognitive representations of the sequence rather than establishing a motoric representation [6].

The present data do not allow a conclusive answer regarding the underlying brain processes yielding the observed behavioral effect. The results may indicate the involvement of the dPMC in motor sequence consolidation as has been argued in a previous study [61] but may also indicate its involvement in recall of the sequence. Since the present data did not provide evidence for a significant relation between reaction times at t2 in sequential trials and reproduction 1, we here would argue in favor of the first hypothesis, the involvement of the dPMC in motor sequence consolidation. Assuming that the dPMC is particularly engaged in the recall of a motor sequence, one would expect that cathodal tDCS should already affect the reproduction performance immediately after training on the SRTT. Since the present data do not support this hypothesis, we here would speculate that cathodal tDCS may have interfered with the brain processes establishing a memory trace allowing subsequent recall but not the recall itself.

### 3.4. Consolidation during Sleep

Converging evidence for the benefit of sleep on consolidation of explicitly learned motor skills exists (reviewed in [18]) but has been shown also for attentive implicit motor sequence learning (reviewed in [10]). Performance improvement after overnight sleep has been shown to be associated with reduced brain activation in prefrontal, premotor and parietal areas suggesting sleep-dependent reorganization [86]. Evidence for the involvement of the PMC for sleep-related consolidation of an implicitly learned sequence exists [79]. The authors found improved reaction times during execution of a newly learned SRTT, when anodal tDCS was applied during rapid-eye movement (REM) sleep and the amount of REM-sleep was correlated with improvement in performance rates [87]. Those findings agree well with the observation that LTP in the hippocampus can be induced during REM- but not during slow-wave sleep [88] supporting the relevance of REM-sleep for synaptic stabilization. On the other hand, in another study, performance improvement was correlated with the amount of stage 2 non-REM sleep [89]. Thus, the exact functional significance of different sleep states (REM vs. Non-REM) for different types of learning (implicit vs. explicit) has yet to be solved.

### 3.5. Limitations

One of the most critical issues that needs to be considered when using tDCS is its relatively low spatial focality (reviewed for example, in [9,56]. This raises the problem of co-stimulation of adjacent and functionally connected brain areas like M1, SMA and the SPC. In a previous study it has been shown that anodal tDCS applied to the left posterior parietal cortex yields a diffuse rise of cortical excitability expanding to the right hemisphere [90]. The simulation data support this finding and although the strongest electrical field was found anterior to the active electrode, we cannot exclude the possibility that the observed behavioral effects are due to functional effects on remote areas. In particular, since we did not control for possible effects of tDCS on M1-excitability and evidence for long-lasting effects on subcortical brain areas in animals exists [91,92,93]. Consequently, the present data should be seen as a piece of evidence for a possible contribution of the left dPMC in explicit motor sequence learning with the right hand. In particular, it is stressed that the effects observed in the present study does not allow any conclusions regarding the functional role of the right dPMC for motor sequence learning with the non-dominant left hand nor regarding brain processes associated with this type of task in left-handers. Another important limitation of tDCS is the fact that the direction of stimulation (i.e., excitatory vs. inhibitory) depends on the direction of current flow in the brain which might vary between brain areas and between participants due to anatomical differences (reviewed for example, in [9]). Finally, the sham stimulation might not be the ideal control condition due to the short stimulation duration.

In the present study, relatively low current intensities were applied. One might speculate that longer stimulation or stimulation at higher intensities would have resulted in more pronounced effects. But, evidence against this hypothesis has been provided by showing that enhancement of tDCS intensity might shift the direction of stimulation effects rather than increasing the efficacy of stimulation [94].

The relatively short sequence of eight items only should be seen as another limiting factor. Immediately after training on the SRTT all participants were able to reproduce at least 6 of 8 sequence items following anodal tDCS and sham stimulation, likely yielding a ceiling effect. In order to prove the hypothesis that anodal tDCS applied to the left dPMC may have the potential to improve explicit motor sequence learning, longer sequences should be implemented to increase the task complexity.

Finally, we interpreted the present findings in terms of tDCS effects on explicit motor sequence learning but we cannot exclude the possibility that cathodal tDCS could have affected motor command execution. Noteworthy, this effect was not evident in random trials.

## 4. Conclusions

The present findings indicate a significant inverse relation between reaction times in sequential trials at the end of the training (t2) and explicit reproduction of the learned sequence after overnight sleep following cathodal tDCS. The data should be seen as first evidence for the hypothesis that cathodal tDCS applied to the left dPMC prior to SRTT training interferes with brain processes enabling explicit retrieval after overnight sleep. Whether this is due to interference with motor sequence consolidation, recall of the motor sequence or motor command execution has yet to be solved. Nevertheless, the present data suggest the left dPMC as one possible target for the application of non-invasive brain stimulation techniques in explicit motor sequence learning with the right hand.

## Figures and Tables

**Table 1:** Transcranial direct current stimulation (tDCS) side effects.

Participants	Reports		
	Sham	Anodal	Cathodal
01	Slightly increased tonicity of jaw muscles	None	Flicker sensation left eye at the end of stimulation
02	Slight tingling below electrodes	Slight tingling below electrodes	Slight itching below frontal electrode
03	None	None	None
04	None	None	None
05	None	None	None
06	None	None	None
07	Slight tingling below electrodes	None	None
08	None	None	None
09	None	Slight tingling below electrodes	Slight tingling below frontal electrode
10	Slight tingling below electrodes	Slight tingling below electrodes	None
11	Warmness below electrodes	None	None
12	None	None	Pulsation below electrodes
13	None	None	Slight tingling below electrodes
14	Slight tingling below electrodes	None	None
15	None	Slight tingling below electrodes	Slight tingling below electrodes
16	None	Slight tingling/itching below electrodes	Slight tingling below electrodes A single twitch of the leg
17	None	None	None
18	None	None	None

**Table 2:** Regression analyses.

**(A) Using Reproduction 1 as Criterion (Number of Correctly Reproduced items)**						
	Predictor	b	*beta*	SE	T	*p*
Sham	Intercept	10.112		2.531	3.995	0.002
	T1 (ms)	−0.001	−0.028	0.006	−0.092	0.928
	T2 (ms)	−0.007	−0.383	0.006	−1.272	0.226
Anodal	Intercept	13.478		3.015	4.470	0.001
	T1 (ms)	−0.015	−0.572	0.009	−1.549	0.145
	T2 (ms)	0.002	0.098	0.008	0.266	0.794
Cathodal	Intercept	5.733		7.806	0.734	0.477
	T1 (ms)	0.008	0.124	0.017	0.433	0.673
	T2 (ms)	−0.008	−0.262	0.009	−0.912	0.380
**(B) Using Reproduction 2 as Criterion (Number of Correctly Reproduced Items)**						
	Predictor	b	*beta*	SE	T	*p*
Sham	Intercept	3.190		4.729	0.675	0.512
	T1 (ms)	0.004	0.112	0.011	0.343	0.737
	T2 (ms)	0.000	0.012	0.011	0.037	0.971
Anodal	Intercept	5.078		4.349	1.168	0.264
	T1 (ms)	−0.005	−0.147	0.014	−0.362	0.723
	T2 (ms)	0.011	0.397	0.012	0.975	0.347
Cathodal	Intercept	16.376		5.314	3.082	0.010
	T1 (ms)	−0.012	−0.229	0.012	−1.002	0.336
	**T2 (ms)**	**−0.015**	**−0.547**	**0.006**	**−2.396**	**0.034**

Note: Significant results are printed in bold.

## Data Availability

The data presented in this study are available on request from the corresponding author. The data are not publicly available due to ethical restrictions.
